# Effect of Additives on the Dissolution Rate of Different Carbonate Rocks by Hydrochloric Acid

**DOI:** 10.1021/acsomega.5c12017

**Published:** 2026-02-13

**Authors:** Ernani Dias da Silva Filho, Guilherme Mentges Arruda, Luan Vinicius Luna dos Santos, Maurício Gabriel Lacerda Mafra, Milton Morais Xavier Junior, Aline Maria Fernandes Galdino da Silva, José Ilton Sarmento Silveira Júnior, Edney Rafael Viana Pinheiro Galvão, Mateus Palharini Schwalbert, Marcos Allyson Felipe Rodrigues

**Affiliations:** † Department of Petroleum Engineering, 28123 Federal University of Rio Grande do Norte, 59064-970 Natal, RN, Brazil; ‡ Department of Geophysic, Federal University of Rio Grande do Norte, 59072-970 Natal, Brazil; § Department of Civil and Environmental Engineering, Federal University of Rio Grande do Norte, 59078-970 Natal, Brazil; ∥ Petrobras, Rio de Janeiro−RJ 20930-040, Brazil

## Abstract

Matrix acidizing is a widely applied technique for stimulating carbonate reservoirs by promoting mineral dissolution and restoring well productivity. In this context, this study aimed to investigate the effect of commercial additives, a corrosion inhibitor and an emulsion preventer, both containing surfactants in their formulations, on the dissolution rate of different carbonate rocks by hydrochloric acid (HCl), comparing the performance of systems with and without additives. Samples of Indiana Limestone, Mount Gambier Limestone, Wisconsin Dolomite, and Bonneterre Dolomite, which exhibit distinct mineralogical compositions and structural characteristics, were analyzed. The samples were characterized using X-ray diffraction (XRD), X-ray fluorescence (XRF), nuclear magnetic resonance (NMR), and porosity measurements. Dissolution experiments were conducted in a batch reactor, allowing the acquisition of kinetic curves for the acid–rock reaction. The results showed that, in the absence of additives, predominantly calcitic rocks exhibited the highest dissolution rates (exceeding 1 g min^–1^), with dissolution kinetics primarily controlled by mass-transfer limitations. In contrast, dolomitic rocks displayed lower reactivity, with rates below 0.1 g min^–1^. The presence of additives significantly retarded the reaction in all rock types, with the most pronounced effect observed for calcitic samples, where the dissolution time increased by more than 18-fold. This behavior can be attributed to foam formation and the reduction of H^+^ availability at the acid–rock interface caused by the surfactants contained in the additives.

## Graphical abstract

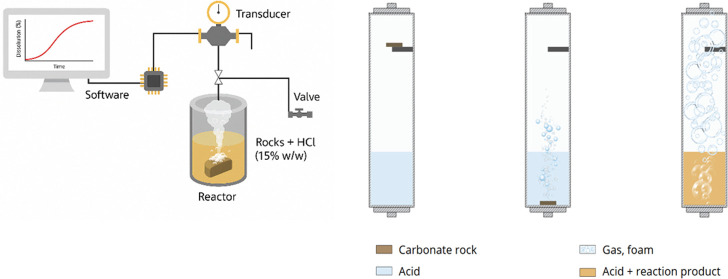

## 1 Introduction

The oil industry faces significant challenges during field operations, such as drilling and completion, among which formation damage is one of the most critical. This phenomenon occurs due to the undesirable invasion of solids and liquids from drilling or completion fluids into the geological formation adjacent to the wellbore. Formation damage may also result from the precipitation of secondary mineral phases, such as sulfates and Fe­(III)-bearing materials, including both amorphous ferrihydrite and crystalline iron oxides.^1,2^ These phases can form due to mineral alteration and from interactions between additive degradation products and formation water. As a result, the permeability of the rocks surrounding the well may decrease, impairing hydrocarbon flow from the reservoir during the production stage.^3,4^ In this scenario, stimulation techniques emerge as alternatives to remove or bypass such damage and, consequently, restore or enhance well productivity.

Among stimulation methods, matrix acidizing stands out as an effective and widely applied approach in carbonate reservoirs. This process is based on the injection of acid solutions, commonly hydrochloric acid (HCl), to dissolve minerals in the rock, thereby creating flow channels and/or increasing pore connectivity. In addition, acid stimulation may contribute to fracture propagation by locally increasing pressure at fracture tips, improving fluid flow pathways and connectivity within the formation.^5–7^ However, the efficiency of this process depends on several factors, including the mineralogical composition of the formation, the properties of the stimulation fluid, and the pressure and temperature conditions of the reservoir.

Carbonate reservoirs are particularly challenging due to their mineralogical diversity, which predominantly involves calcite (CaCO_3_) and dolomite (CaMg­(CO_3_)_2_), as well as silicate impurities and other minerals resistant to HCl that can influence the acid–rock reactivity.^8,9^ This complexity requires a detailed understanding of dissolution kinetics, encompassing both the reaction rates and the mechanisms by which the acid interacts with different mineralogies.

In addition to mineralogical composition, the presence of additives in the stimulation fluid also plays a fundamental role in the dynamics of the reaction. Compounds such as corrosion inhibitors (CI), which may contain surfactants in their composition, are used to protect metallic equipment during acidizing operations.^10,11^ Moreover, these additives can modify physicochemical properties of the acid solution, such as viscosity and surface tension, directly affecting the efficiency of the process.^12^

Understanding the rock–fluid interaction is essential in dissolution kinetics studies, as it enables the prediction of acidizing efficiency and the development of optimized formulations that can reduce operational costs. Here, the present study investigates the influence of commercial additives, a corrosion inhibitor and an emulsion preventer, on the dissolution rate of Indiana Limestone, Mount Gambier Limestone, Wisconsin Dolomite, and Bonneterre Dolomite, which exhibit distinct mineralogical compositions and pore structures. The goal is to provide experimental data that contribute to optimizing the performance of stimulation fluids in complex carbonate environments, supporting the development of more efficient and sustainable solutions for the oil and gas industry.

## 2 Materials and Methods

### 2.1 Materials

To prepare the reactive fluid used in the experiments, the following reagents and components were employed: hydrochloric acid (HCl, 37%, Neon); corrosion inhibitor (CI) and emulsion preventer (EP). Due to confidentiality agreements with the supplier, the detailed composition of these additives cannot be disclosed. The additives used in this study are representative of commercially available products commonly applied in acidizing operations. The corrosion inhibitor consists primarily of a mixture of surfactants, quaternary ammonium salts, alcohols, and aldehydes, which act by reducing metal corrosion and modifying interfacial properties. The emulsion preventer is mainly composed of nonionic surfactants and ethanol, designed to minimize emulsion formation between the acid solution and hydrocarbon phases. This description is provided to clarify the expected physicochemical behavior of the additives during the acid–rock interaction. Commercial carbonate rocks of the Indiana Limestone (IL), Mount Gambier Limestone (MG), Wisconsin Dolomite (WL), and Bonneterre Dolomite (BT) types were supplied by Kocurek Industries, Inc. (Texas). All aqueous solutions were prepared using distilled water.

In the following sections, the abbreviations used are as follows: NA refers to the acid solution without additives, and CA to the acid solution with additives. Combined labels (e.g., ILNA, ILCA, BTNA, BTCA) identify each rock type tested under the corresponding acid system.

#### 2.1.1 Preparation of the Acid Fluid

Experiments were conducted with and without additives. For all tests, concentrated HCl (37%) was diluted to 15 wt % using distilled water and weighed on a precision balance (model AD5002, Marte Científica). In the tests containing additives, the corrosion inhibitor was added to reach 0.5% (v/v) of the final solution, followed by the addition of the emulsion preventer to reach 0.2% (v/v).

#### 2.1.2 Preparation of Rock Samples

Core plugs measuring 6 in. in length and 1.5 in. in diameter were sectioned into discs. Each disc was subsequently cut in half, and the resulting pieces (weighing approximately 2 g) were used in the experiments, as presented in Figure 1. The samples were carefully processed to ensure similar dimensions and consistent mass. Each specimen underwent detailed visual inspection to ensure that its surface was free from defects that could interfere with the interaction between the rock and the reactive fluid.

**Figure 1:**
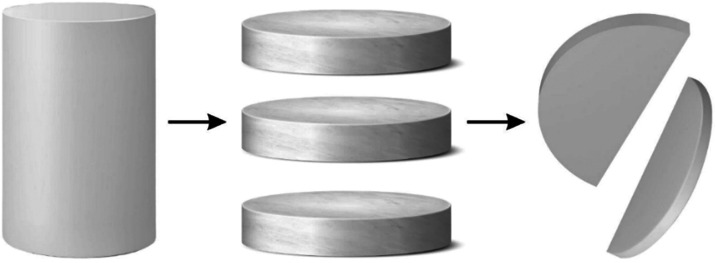
Example of the preparation of the samples used in the reactor.

### 2.2 Characterization of Acid Solutions

#### 2.2.1 Density

The density of the acid solution was measured to assess the effect of additives on its physicochemical properties. Measurements were performed in triplicate at 25 °C using a digital densimeter (Anton Paar). The mean value obtained represents the density of the acid solution containing additives (CA), which was used to verify whether the additives induced any significant change that could influence practical parameters such as hydrostatic pressure during acid injection in field operations. In addition, the measured density served to convert kinematic viscosity values obtained from the capillary viscometer into dynamic viscosity.

Since the physicochemical properties of aqueous HCl solutions are well established in the literature, the density of 15 wt % HCl without additives was not measured experimentally but estimated numerically by interpolation of tabulated data.

#### 2.2.2 Viscosity

Viscosity analyses were performed to investigate the impact of additives on the physical properties of aqueous HCl. This property directly influences the effect of the fluid to penetrate rock pores and the diffusion resistance of H^+^ ions toward the rock surface, resulting in a decrease in the carbonate dissolution rate at higher viscosities.^13^ Measurements were carried out using a Cannon-Fenske capillary viscometer partially immersed in a thermostatic bath at 25 °C. Viscosity was calculated using (eq 1)­

1 $$\mu_{S}=\mu_{w}\frac{\rho_{s}t_{s}}{\rho_{w}t_{w}}$$

where μ_s_, ρ_s_ and *t*_s_ correspond to the viscosity, density, and efflux time of the acid solution, respectively, and μ_w_, ρ_w_ and *t*_w_ refer to the same parameters for distilled water. Each measurement was repeated in triplicate to ensure precision.

#### 2.2.3 Surface Tension

The surface tension of the 15 wt % HCl solutions, with and without the addition of additives, was determined using a Krüss K20 tensiometer at 25 °C under controlled laboratory conditions. The analyses were conducted for two experimental conditions: pure 15 wt % HCl and 15 wt % HCl containing both the corrosion inhibitor and emulsion preventer at their respective standard concentrations.

### 2.3 Rock Characterization

#### 2.3.1 Mineralogical Composition

For mineralogical characterization of the rock samples, one representative sample from each group (IL, MG, WL, and BT) was ground into fine powder. The analyses were performed using X-ray diffraction (XRD) and X-ray fluorescence (XRF) techniques. The XRD patterns were obtained using a Bruker D2 Phaser benchtop diffractometer equipped with a Lynxeye detector. Measurements followed the Bragg–Brentano configuration (θ-2θ), covering an angular range of 10° to 80°, with a step size of 0.02° and a scanning rate of 2° min^–1^. The radiation used was Cu Kα (λ = 1.54 Å), and all analyses were conducted at ambient temperature (25 °C). The data were processed using the ICSD database and refined by the Rietveld method.^14^

Elemental composition was determined by XRF using a BRUKER S2 PUMA – SERIES II spectrometer with a palladium (Pd) tube, operating at a maximum power of 50 W, voltage up to 50 kV, and current up to 1 mA, coupled with a HighSense Silicon Drift Detector (HighSense SDD).

#### 2.3.2 Porosity

Effective porosity measurements were performed using six randomly selected samples for each group. The dry samples were weighed, saturated with a brine solution of known density, and then weighed again. Knowing the sample mass before and after saturation (*m*_dry_ and *m*_sat_, respectively) and the brine density (ρ_brine_), the pore volume (*V*_p_) and effective porosity (Φ_eff_) were calculated using eqs 2, 3, and 4, assuming complete saturation (*S*_brine_ (%) = 100) and using the total sample volume (*V*_t_) previously determined. In this context, effective porosity refers to the interconnected pore volume accessible to fluid flow.

2 $$V_{\mathrm{brine}}=\frac{\left(m_{\mathrm{sat}}-m_{\mathrm{dry}}\right)}{\rho_{\mathrm{brine}}}$$

3 $$V_{p}=\frac{V_{\mathrm{brine}}}{S_{\mathrm{brine}}}$$

4 $$\Phi_{\mathrm{eff}}=\frac{V_{p}}{V_{t}}$$

For fully saturated samples *V*_p_ = *V*_brine_.

#### 2.3.3 Nuclear Magnetic Resonance (NMR)

Low-field nuclear magnetic resonance (NMR) was employed to evaluate the pore-size distribution and pore-throat radii of the samples. It should be noted that the pore-size distribution and pore-throat radii discussed in this study represent only the interconnected pore network accessible to fluids, as noninterconnected pores are not captured by the measurement method and are therefore assumed not to contain mobile fluid. This technique is based on the effect of hydrogen nuclei in porous media to relax after being subjected to a magnetic field sequence, allowing the estimation of properties such as porosity and pore-size distribution.^15^

Measurements were conducted using a MesoMR12–060H–I instrument (Niumag) to obtain the transverse relaxation time (*T*_2_) according to eq 5, using the Carr–Purcell–Meiboom–Gill (CPMG) pulse sequence.^16,17^ When the pulse intervals in the CPMG sequence are short, the relaxation times associated with bulk and diffusion fractions can be neglected, leaving only the surface relaxation component, as expressed in eq 6.^18^ This assumption is valid if the surface relaxivity (ρ_2_) remains uniform along the pore walls.^19^

5 $$\frac{1}{T_{2}}=\frac{1}{T_{2\;\mathrm{bulk}}}+\frac{1}{T_{2\;\mathrm{surface}}}+\frac{1}{T_{2\;\mathrm{diffusion}}}$$

6 $$\frac{1}{T_{2\;\mathrm{surface}}}=\rho_{2}\frac{S}{V}\approx \frac{1}{T_{2}}$$

From the relaxation curve, a distribution of relaxation times can be obtained. If the pores have similar geometries, larger pores present smaller surface-to-volume ratios (S/V), resulting in longer relaxation times. Thus, a correlation can be established between *T*_2_ values and pore or pore-throat sizes, assuming a constant surface relaxivity ρ_2_.

In this study, ρ_2_ was assumed to be the same for all samples, allowing the relationship between the *T*_2_ distribution and pore-throat radius to be established. The pore-throat radius was calculated according to the Godefroy et al. Model^20^ shown in eqs 7 and 8. This model was developed by correlating NMR transverse relaxation times with mercury intrusion porosimetry data. Larger pore throats correspond to larger connected pores, indicating that greater pore-throat radii are associated with higher permeability and fluid accessibility.

7 $$r=0.0033{T_{2}}^{0.372}\left(T_{2}<30\;\mathrm{ms}\right)$$

8 $$r=0.0003{T_{2}}^{1.840}\left(T_{2}\geq 30\;\mathrm{ms}\right)$$

### 2.4 Dissolution Kinetics of Carbonate Rocks

To evaluate the influence of the additives (CI and EP) on the dissolution rate of different carbonate rocks by hydrochloric acid (HCl), a batch-type reactor was developed. The system operates as a closed vessel equipped with a pressure transducer (maximum capacity of 345 kPa, or 50 psig) and a relief valve. All experiments were conducted at a controlled temperature of 25 °C to ensure reproducibility and allow direct comparison with other physicochemical analyses. Figure 2(a) presents the experimental setup used for the dissolution tests.

**Figure 2:**
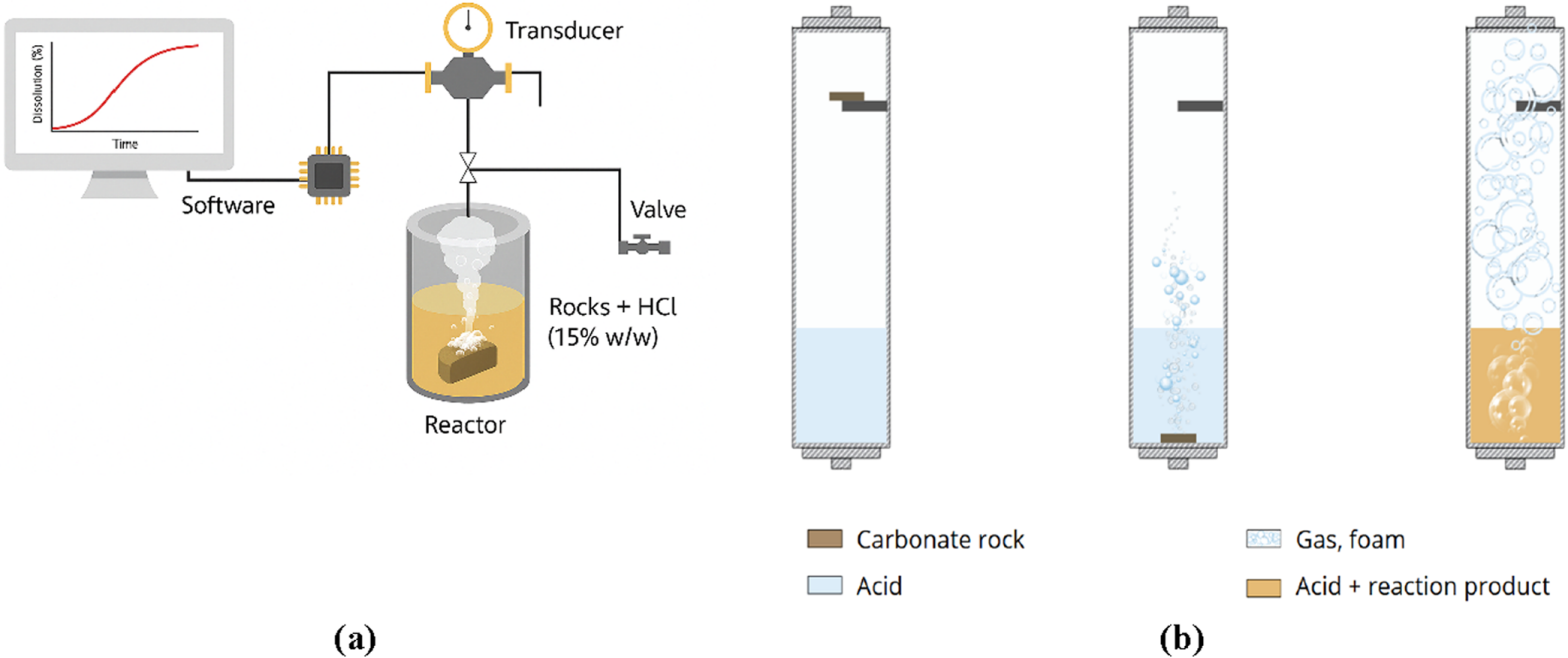
Schematic diagram of the complete reactor system used in the dissolution tests (a) and representation of the sample dissolution inside the reactor (b).

The system is integrated with a Data Acquisition (DAQ) module that records pressure data and transmits it to custom-developed monitoring software. The software generates real-time pressure variation curves throughout the reaction. When the rock sample meets the acid, dissolution of calcite (eq 9) and dolomite (eq 10) occurs, producing CO_2_, water, and salt. During the reaction, CO_2_ release increases the internal pressure until the acid–rock reaction reaches completion, as indicated by the cessation of CO_2_ generation and stabilization of the system pressure.

9 $$\mathrm{CaC}{O_{3}}_{\left(s\right)}+2\mathrm{HC}l_{\left(\mathrm{aq}\right)}\to \mathrm{CaC}{l_{2}}_{\left(\mathrm{aq}\right)}+{CO_{2}}_{\left(g\right)}^{↑}+H_{2}O_{\left(l\right)}$$

10 $$\mathrm{MgCa}(C{O_{3})}_{2\left(s\right)}+4\mathrm{HC}l_{\left(\mathrm{aq}\right)}\to \mathrm{CaC}{l_{2}}_{\left(\mathrm{aq}\right)}+\mathrm{MgC}{l_{2}}_{\left(\mathrm{aq}\right)}+2{CO_{2}}_{\left(g\right)}^{↑}+{2H}_{2}O_{\left(l\right)}$$

The experiment was designed to ensure that the maximum pressure did not exceed 276 kPa (40 psig). This pressure range was maintained for all experiments to guarantee comparability among results. Additionally, data were normalized in terms of dissolution rate (%), enabling direct comparison across tests regardless of pressure values. The reactor volume was set to 290 mL, and a volume of acid solution (100 mL) was used in excess to minimize concentration variation during dissolution.

Inside the reactor, the acid was added first, and the rock sample was positioned on a support, as shown in Figure 2(b). The lid and relief valve were closed to seal the system, and the monitoring software was initiated. To start the reaction, the system was mechanically agitated to ensure contact between the sample and the acid solution. The pressure evolution was monitored until stabilization, indicating that all carbonate had dissolved and CO_2_ generation had ceased.

Finally, the recorded data were analyzed to generate dissolution curves as a function of time, allowing comparison of HCl performance with and without additives across the different rock types.

## 3 Results and Discussion

### 3.1 Fluid Properties

Since the physicochemical properties of the acid fluid directly influence the dissolution kinetics of carbonate rocks, the density, viscosity, and surface tension of the solutions were characterized to support the subsequent analyses.

#### 3.1.1 Density and Viscosity of the Acid Fluid

The density of the acid solution was measured to assess the impact of additives on its physicochemical properties. Measurements were performed in triplicate for four batches of the same acid formulation (15 wt % HCl with additives) using a digital densimeter (Anton Paar) at 25 °C. For each batch, three readings were taken and averaged, and the overall mean of the four batches was considered the final density of the additive-containing acid solution.

For the 15 wt % HCl solution without additives, density was not measured experimentally but interpolated from well-established literature data. The results are presented in Table 1.

**Table 1:** Density of Four Different Batches with the Same Acid Composition Containing Additives

	measured density (g/mL)			
test number	sample 1	sample 2	sample 3	sample 4
1	1.07137	1.07133	1.07202	1.07179
2	1.07137	1.07133	1.07203	1.07179
3	1.07137	1.07134	1.07203	1.07179
average	1.07137	1.07133	1.07203	1.07179
definitive result	1.07161 ± 0.00034			

Based on the results, the density of the 15 wt % HCl solution did not show significant variation after the addition of additives. The measured values were very close to the reference density reported for 15 wt % HCl in the literature,^21^ with relative deviations below 0.1%, indicating that the additives did not substantially alter this physicochemical property.

For viscosity calculations, based on eq 1, the standard value of 0.89 cP was adopted for the viscosity of water at 25 °C.^22^ Efflux times were measured in triplicate to ensure experimental reproducibility. To improve accuracy, the propagation of uncertainty was calculated based on the standard deviations of efflux times and densities. The results are summarized in Table 2.

**Table 2:** Viscosity Values Obtained for Each Fluid Using a Cannon–Fenske Viscometer and Corresponding Densities

	measured time (s)		
test number	water	HCl	HCl + additives
1	78	86	90
2	75	87	90
3	77	88	91
density (g/mL)	0.99825	1.07049	1.07203
viscosity (cP)	1.0	1.216 ± 0.016	1.261 ± 0.015

The results indicate that the viscosity of the 15 wt % HCl solution remained nearly unchanged after the addition of additives. The measured values are consistent with those reported in the literature for this acid concentration.^21^

#### 3.1.2 Surface Tension

The surface tension measurements of the acid fluid, conducted at 25 °C, are presented in Table 3.

**Table 3:** Surface Tension Obtained Using a Krüss K20 Tensiometer^a^

	volumetric concentration (% v/v)			
acid system	HCl 15 wt %	corrosion inhibitor	emulsion preventer	surface tension (mN/m)
NA	100	0	0	66.01 ± 0.09
CA	99.3	0.5	0.2	31.34 ± 0.06

a NA: Without additives; CA: Containing additives.

The results in Table 3 show that the combined addition of the corrosion inhibitor and emulsion preventer significantly reduced the surface tension of the acid solution, from 66.01 ± 0.09 mN/m in the pure acid to 31.34 ± 0.06 mN/m in the additive-containing fluid. This reduction is attributed to the presence of surfactants in the formulation, which tend to adsorb at the interface and decrease surface tension. Although direct contact tests with oil were not conducted in this study, the literature reports that lowering the surface tension can favor emulsion formation during acidizing processes. Depending on the ratio between the acid fluid and the formation oil, this may enhance the stability of unwanted emulsions, potentially compromising treatment efficiency.^23,24^

### 3.2 Rock Properties

#### 3.2.1 Mineralogical Composition

The XRD analysis of the IL, MG, WL, and BT samples revealed distinct diffraction peaks, as shown in Figure 3, confirming the dominant minerals in each rock type. For the IL and MG samples, the most representative peaks were observed at 2θ angles of 23.06°, 29.46°, 36°, 39.44°, 43.2°, 47.5°, 48.58°, and 57.46°, corresponding to the crystallographic planes of calcium carbonate (CaCO_3_). These results are consistent with previously published data and established standards.^25^ The crystalline structures were refined using the Rietveld method in the MAUD software.^26^ The refined parameters indicated a hexagonal structure, belonging to the *R*3̅*C* space group, with *a* = 4.98 Å, *c* = 17.04 Å, and γ = 120°. When compared with the data reported by Antao and Hassan,^27^ these values confirmed the high-purity calcitic composition of the IL and MG samples, with no secondary mineral phases detected.

**Figure 3:**
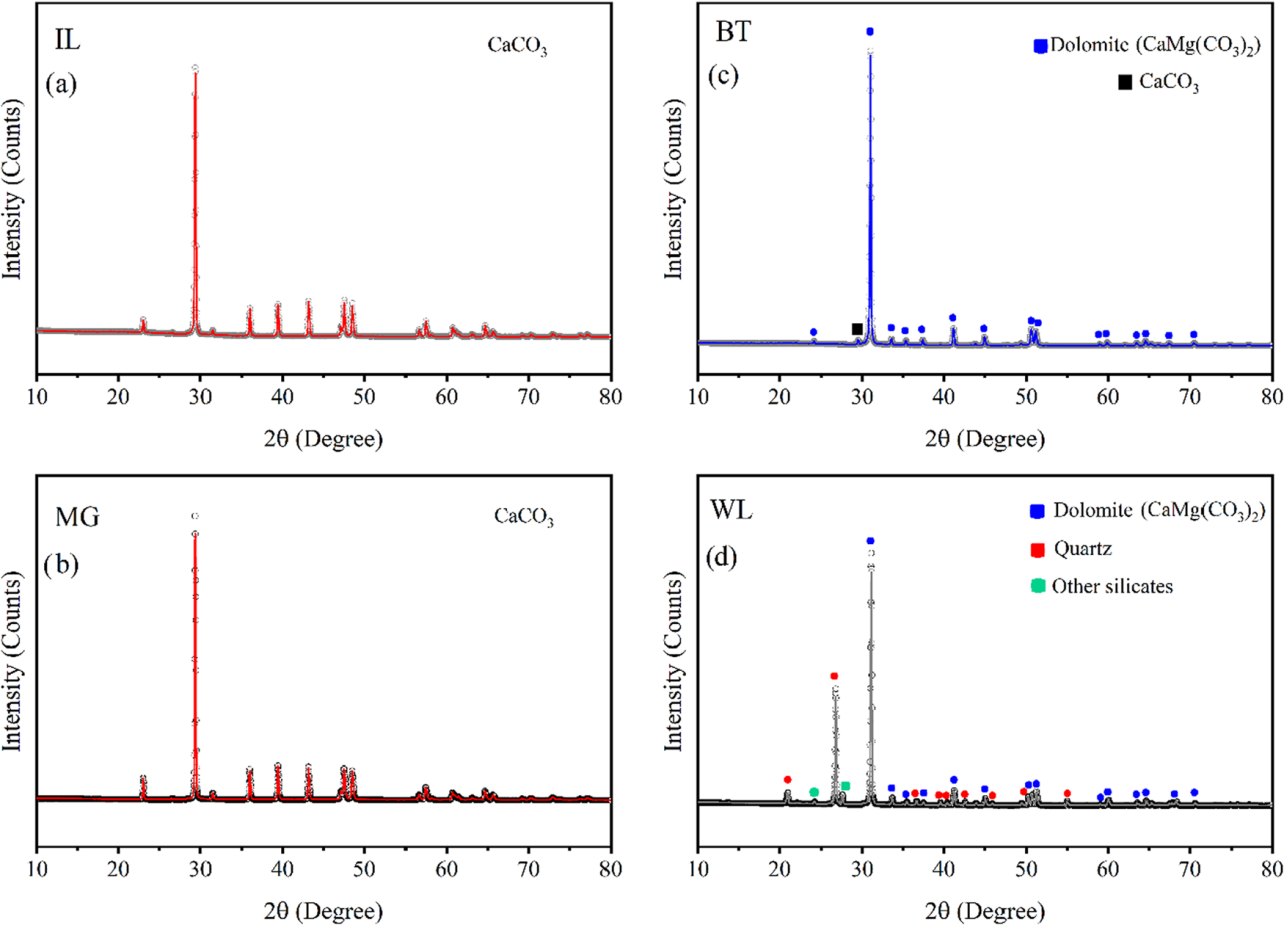
Rietveld refinement profiles of XRD data for carbonate rocks: (a) IL: Indiana Limestone, (b) MG: Mount Gambier Limestone, (c) BT: Bonneterre Dolomite, and (d) WL: Wisconsin Dolomite. Dots represent experimental data and the solid line corresponds to the Rietveld refinement fit.

In contrast, the BT and WL samples displayed diffraction peaks characteristic of dolomite, with 2θ values at 24.2°, 31.02°, 33.7°, 35.5°, 37.5°, 41.3°, 45.1°, 50.7°, 51.22°, 59°, 60°, 63.58°, 64.68°, 68.42°, and 70.66°.^28,29^ The BT sample showed a dominant dolomite peak at 31.02° along with a minor peak at 29.46°, indicating a small calcite presence. The WL sample, in addition to dolomite peaks, exhibited distinct quartz reflections at 2θ = 21°, 26.76°, 36.68°, 39.62°, 40.42°, 42.64°, 45.94°, 50.26°, and 55°,^30^ confirming the presence of silicate phases.

The X-ray fluorescence (XRF) results confirmed the high calcium content of the samples. IL and MG presented high-purity calcitic compositions, with 98.58% and 95.23% CaO, respectively. In contrast, the WL and BT samples contained significant amounts of both CaO and MgO, indicating their dolomitic nature (CaMg­(CO_3_)_2_), whose dissolution is described by the reaction in eq 10. The main chemical compositions of the carbonate rock samples are summarized in Table 4.

**Table 4:** Chemical composition (wt%) of IL: Indiana Limestone, MG: Mount Gambier, WL: Wisconsin Dolomite, and BT: Bonneterre Dolomite

	chemical composition (wt %)			
oxide	Indiana Limestone	Mount Gambier	Wisconsin Dolomite	Bonneterre
CaO	98.58	95.23	40.64	60.80
SiO_2_	0.280	0.980	35.11	2.160
MgO	-	-	20.45	28.64
Na_2_O	-	2.140	-	2.710
K_2_O	0.400	0.300	0.740	0.160
Al_2_O_3_	0.300	0.420	2.380	1.030
SrO	0.270	0.060	-	-
Fe_2_O_3_	-	0.460	0.300	3.490
MnO	-	0.030	0.030	0.560
Cl	-	-	-	-
others	0.170	0.380	0.350	0.450
total	100	100	100	100

The WL sample also exhibited a notable SiO_2_ content, likely related to quartz and other silicate minerals, which can reduce reactivity toward HCl compared to high-purity dolomites. In contrast, the BT sample showed a much lower silica concentration, indicating fewer silicate impurities.

Overall, the XRD and XRF results revealed significant mineralogical differences between the analyzed rocks, which are expected to influence their physical and chemical properties and consequently, their reactivity and dissolution rate in acid media. These aspects are further discussed in the following sections.

#### 3.2.2 Porosity

The calculated porosities are presented in Table 5, including the mean and deviation for each rock type.

**Table 5:** Porosity of Each Rock Sample and Average Porosity for Each Group^a^

	porosity (%)			
sample	IL	MG	WL	BT
1	16.9	50.5	10.3	14.2
2	17.2	50.9	10.2	14.4
3	20.0	49.1	10.2	14.3
4	19.0	49.7	9.4	14.9
5	17.6	47.9	9.7	15.0
6	20.0	48.6	9.6	14.0
average	18.3 ± 1.4	49.4 ± 1.1	10.0 ± 0.4	14.4 ± 0.4

a IL: Indiana Limestone; MG: Mount Gambier Limestone; WL: Wisconsin Dolomite; BT: Bonneterre Dolomite.

Differences in average porosity among the rock groups directly affect the acid–rock interaction observed in reactor experiments. Highly porous samples, such as the MG group (49.4 ± 1.1%), have more open structures. This may facilitate acid penetration and increase the reactive surface area, accelerating dissolution. This behavior agrees with previous studies reporting that higher porosity enhances acid–rock contact area, resulting in faster dissolution rates.^31,32^ Conversely, rocks with lower porosity, such as the WL group (10.0 ± 0.4%), restrict acid access to internal pore spaces, leading to slower reaction kinetics. The IL group, with intermediate porosity (18.3 ± 1.4%). In general, porosity is a key structural factor influencing acid–rock reactivity. However, total porosity alone does not describe pore-size distribution, which plays a decisive role in controlling acid access to reactive surfaces. It should be emphasized that effective porosity, rather than total porosity alone, together with pore-throat size distribution, plays a decisive role in controlling fluid accessibility and acid–rock interaction. Therefore, NMR analyses were performed to further characterize the pore network of each rock type.

#### 3.2.3 Nuclear Magnetic Resonance (NMR)

From the transverse relaxation times (*T*_2_) obtained in the nuclear magnetic resonance (NMR) experiments, the pore radii were calculated according to the model proposed by Han et al.^33^ The pore throat distribution for each rock type is shown in Figure 4.

**Figure 4:**
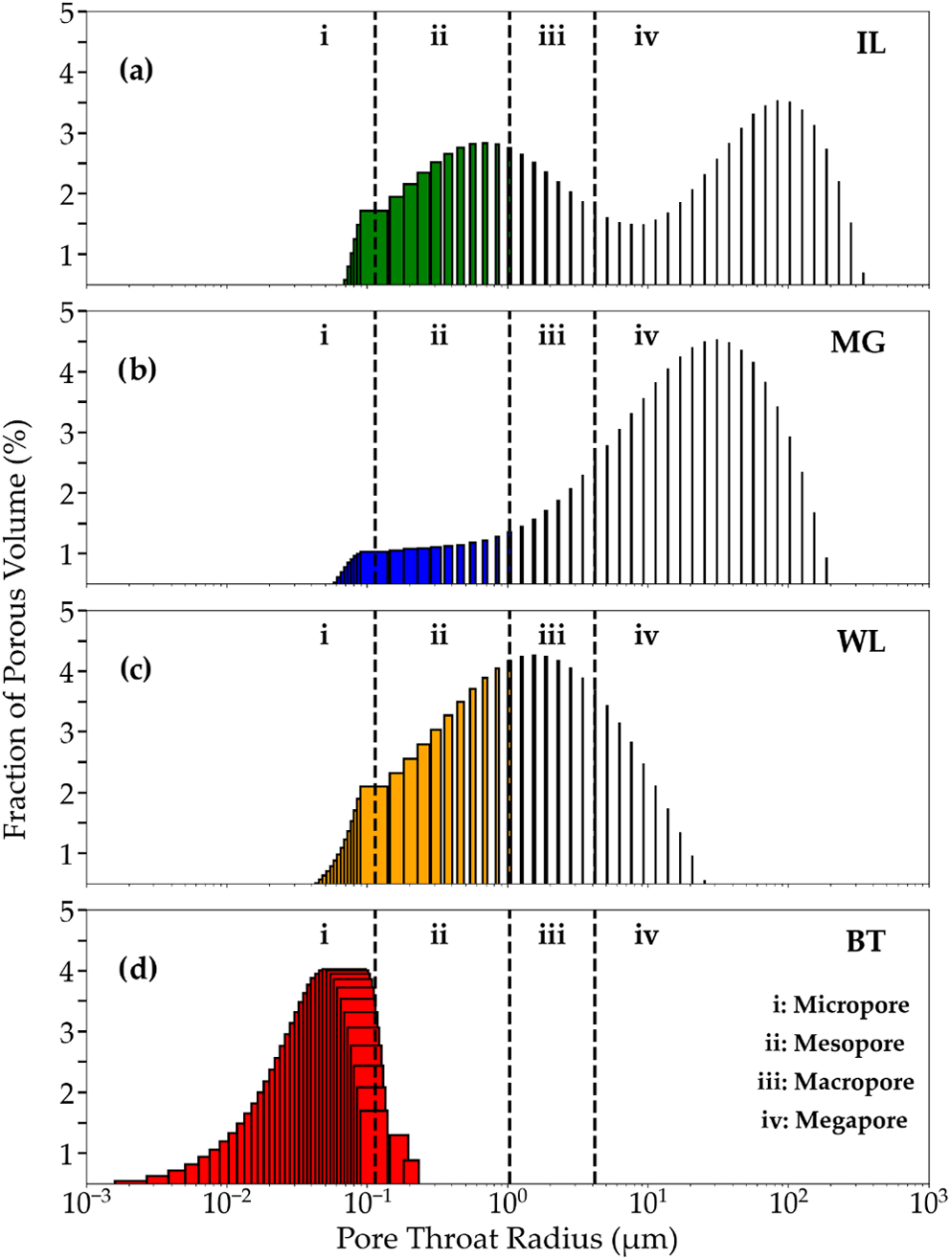
Pore throat distribution of each rock type: (a) Indiana Limestone (IL), (b) Mount Gambier Limestone (MG), (c) Wisconsin Dolomite (WL), and (d) Bonneterre Dolomite (BT). The dashed vertical lines indicate the pore size classification as micropores (i), mesopores (ii), macropores (iii), and megapores (iv).

In addition, the fraction of micropores, mesopores, macropores, and megapores relative to the total pore volume of each sample was quantified using the method described by Ge et al.,^34^ and the results are summarized in Table 6.

**Table 6:** Fraction of Pore Volume Corresponding to Each Pore-Size Category for the Different Carbonate Rocks^a^

	fraction of porous volume (%)			
pore size type	IL (%)	MG (%)	WL (%)	BT (%)
micropores	7.57	8.36	19.44	98.65
mesopores	25.58	11.60	33.30	1.35
macropores	15.26	13.47	28.51	0.00
megapores	51.58	66.56	3.42	0.00

a IL: Indiana Limestone; MG: Mount Gambier Limestone; WL: Wisconsin Dolomite; BT: Bonneterre Dolomite.

The IL rock (Figure 4a) exhibited a broad pore-size distribution, with most of its pore volume classified as megapores (51.58%). Similarly, the MG sample (Figure 4b) showed an even higher concentration in the megapore range (66.56%). The presence of large pores contributes to higher effective porosity. When combined with the high total porosity (discussed in Section 3.2.3), it can provide an even greater fluid storage capacity.^35,36^ This combination of high total porosity and abundant megapores might improve acid accessibility, which in turn increases the effectively exposed reactive surface area, thereby enhancing the acid–rock interaction and leading to faster reaction kinetics, as will be discussed in Section 3.3.

According to Zhang et al.,^37^ pore-size distribution plays a key role in the reaction rate between acid and rock, with larger pores providing more effective flow channels and accelerating the dissolution process. In contrast, the WL (Figure 4c) and BT (Figure 4d) samples exhibited more uniform pore-size distributions. The WL sample contained a wider range of pore-size classes, while the BT sample had its porosity almost entirely restricted to the micropore range, indicating limited acid accessibility and slower dissolution behavior.

### 3.3 Dissolution Kinetics of Carbonate Rocks

The dissolution rate of each rock type in 15 wt % HCl without additives, as determined from the reactor experiments, showed significant variations (Figure 5). For higher accuracy, it was assumed that the rock samples were completely dissolved when the system reached 99% of the maximum pressure recorded by the transducer.

**Figure 5:**
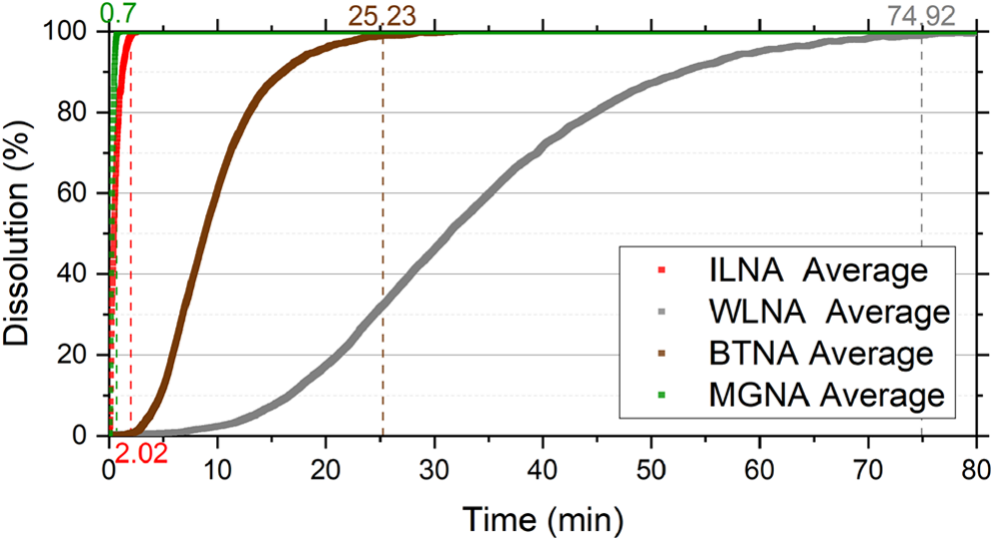
Dissolution reaction curves of IL, BT, and WL rocks in additive-free acid solution.

Because Indiana Limestone (IL) and Mount Gambier (MG) are predominantly composed of CaCO_3_, the reaction between 15 wt % HCl (NA) and these samples (ILNA and MGNA, respectively) under the experimental conditions is limited by mass transfer of H^+^ ions to the rock surface.^38^ Thus, when H^+^ ions from the acid meet the rock surface, they react almost instantaneously. In the ILNA experiment, dissolution was completed in an average time of 2.02 min, whereas in MGNA, it occurred in only 0.70 min. These results are consistent with the porosity and NMR data previously discussed, which showed that the MG group displays not only higher total porosity but also a greater proportion of megapores. This pore structure facilitates acid penetration into the matrix and improves accessibility to reactive surfaces, thereby increasing the effectively exposed surface area and accelerating the dissolution kinetics, even among rocks with similar mineralogies, as also reported by.^39^

The other rock samples are predominantly dolomitic and contain additional impurities such as silicates, potentially contributing to longer dissolution times. Based on the stoichiometry of calcite (eq 9) and dolomite (eq 10) dissolution, dolomite requires a larger amount of acid to dissolve. Furthermore, under these conditions, dolomite dissolution is known to be surface-reaction limited,^40^ which explains the longer reaction times observed for WLNA and BTNA samples, 74.92 and 25.23 min, respectively. The presence of minerals such as quartz in WL may also account for its lower dissolution rate, since quartz is resistant to HCl solutions. The presence of quartz reduces the mineral surface effectively available for acid–rock interaction, limiting the overall dissolution process.

The addition of the emulsion preventer and corrosion inhibitor to the reactive fluid significantly altered the reaction rates, considerably for IL and MG, moderately for WL, and slightly for BT, as shown in Figure 6. During the reaction, the additives, which contain surfactants in their composition, generate a foam that hinders the migration of CO_2_ from the surface of the sample to the bulk solution and, consequently, the transport of new H^+^ ions from the bulk solution to the surface.^12^ In addition, the interaction between the micellar structures of the surfactants and the rock surface can form a protective film, reducing rock exposure to HCl and slowing the reaction.^41,42^ Therefore, a delay in the acid–rock reaction was observed in all mineralogies due to the presence of additives.

**Figure 6:**
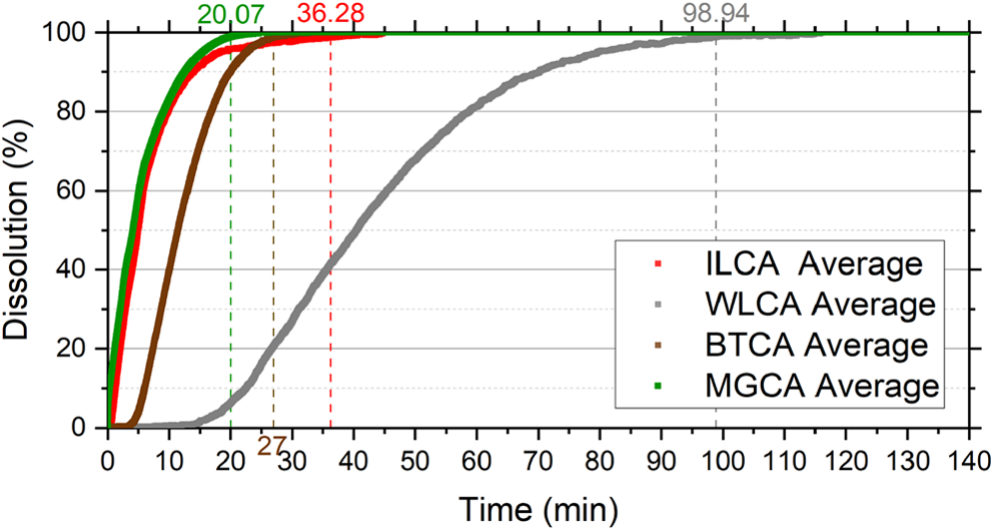
Dissolution reaction curves of carbonate rocks in acid solution containing additives (CA): ILCA (Indiana Limestone), MGCA (Mount Gambier Limestone), BTCA (Bonneterre Dolomite), and WLCA (Wisconsin Dolomite). CA refers to 15 wt % HCl containing corrosion inhibitor and emulsion preventer. Curves represent average values obtained from duplicate experiments.

The ILCA sample exhibited a final dissolution time of 36.28 min, an increase of about 18 times compared to ILNA. The MGCA required 20.07 min, representing an increase of 28 times compared to MGNA. The WLCA and BTCA samples were dissolved in 98.94 and 27 min, respectively, corresponding to increases of 1.3 and 1.07 times compared to WLNA and BTNA. These results confirm the retardation effect caused by the additives, corroborating the mechanisms discussed earlier.

Among the tested rocks, IL and MG were more affected by the presence of additives than the dolomitic samples (WL and BT), consistent with previously reported differences in carbonate dissolution kinetics.^38,43^ It is believed that in systems where H^+^ transport to the reactive surface is the rate-limiting step, the foam formation induced by surfactants complicates the mass transfer, further reducing reaction efficiency.

It is important to note that the experiments were conducted under low-pressure conditions, which differ significantly from typical downhole pressures that can exceed tens of MPa (thousands of psi). At low pressures, the CO_2_ produced during the acid–rock reaction remains in the gaseous phase, occupying a large volume within the system. In contrast, under reservoir conditions, more CO_2_ remains dissolved in the aqueous phase or appears as a supercritical phase, with much higher density and significantly reduced free volume. This contrast can affect both dissolution kinetics and overall reaction dynamics in field conditions. A more representative experimental alternative would be the use of a rotating disk apparatus, which could yield data closer to downhole conditions. This aspect represents a relevant suggestion for future studies.

At reservoir temperature and pressure, the CO_2_ generated during HCl–carbonate reactions is less likely to evolve as a large free gas phase, as observed under atmospheric conditions. Instead, a significant fraction of CO_2_ may remain dissolved in the aqueous phase and/or exist as a dense supercritical phase. This change in CO_2_ phase behavior can modify dissolution kinetics by reducing gas-volume expansion, altering interfacial mass-transfer conditions, and shifting carbonate–bicarbonate equilibria in the aqueous phase.^44^ In additive-free systems, elevated temperature generally accelerates carbonate dissolution by increasing intrinsic reaction rates and diffusion, whereas high pressure suppresses extensive gas-phase CO_2_ formation. In the presence of surfactant-based additives, the retardation mechanisms observed at ambient conditions, such as foam formation and the development of interfacial films that limit H^+^ availability at the rock surface, may be altered under HPHT conditions. Elevated temperature may reduce foam stability and weaken surfactant adsorption, while dense or supercritical CO_2_ phases may affect interfacial resistance and CO_2_–surfactant interactions. Consequently, the relative influence of temperature, pressure, and additives is expected to depend on mineralogy and on whether the system is mass-transfer- or surface-reaction-controlled.^45–47^

Figures 7, 8, 9, and 10 show the dissolution reaction profiles as a function of time, comparing the effect of acid with and without additives for each mineralogy. It can be observed that, in addition to the significant difference in dissolution times among mineralogies, the additives also act as reaction retarders.

**Figure 7:**
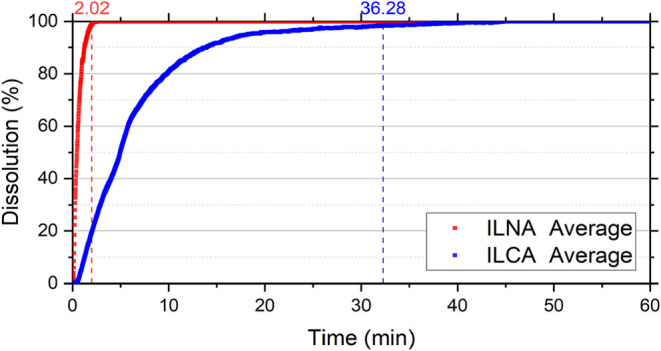
Dissolution reaction curves of Indiana Limestone in 15 wt % HCl with and without additives as a function of time. ILNA corresponds to Indiana Limestone reacted with 15 wt % HCl without additives, and ILCA corresponds to Indiana Limestone reacted with 15 wt % HCl containing corrosion inhibitor and emulsion preventer. Curves represent average values obtained from duplicate experiments.

**Figure 8:**
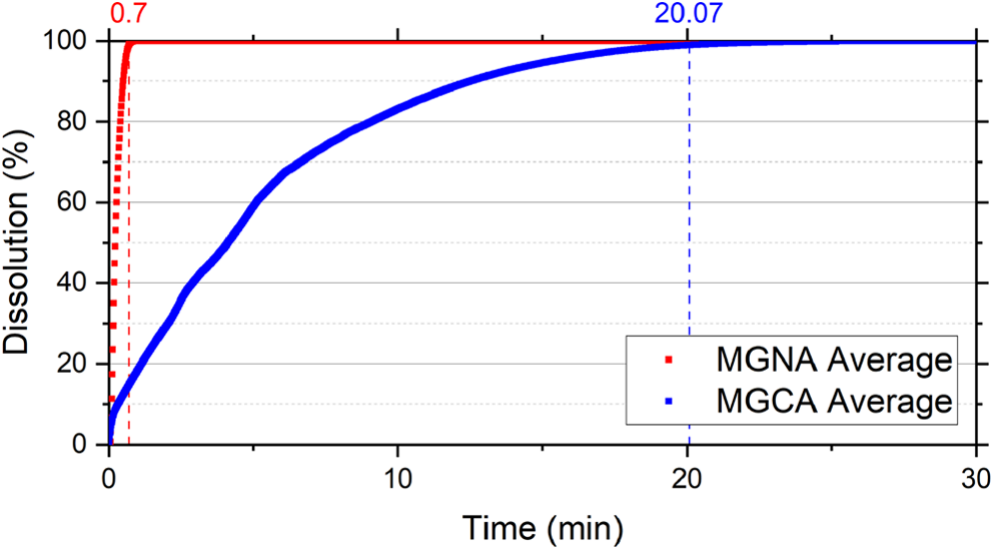
Dissolution reaction curves of Mount Gambier Limestone in 15 wt % HCl with and without additives as a function of time. MGNA corresponds to Mount Gambier Limestone reacted with 15 wt % HCl without additives, and MGCA corresponds to Mount Gambier Limestone reacted with 15 wt % HCl containing corrosion inhibitor and emulsion preventer. Curves represent average values obtained from duplicate experiments.

**Figure 9:**
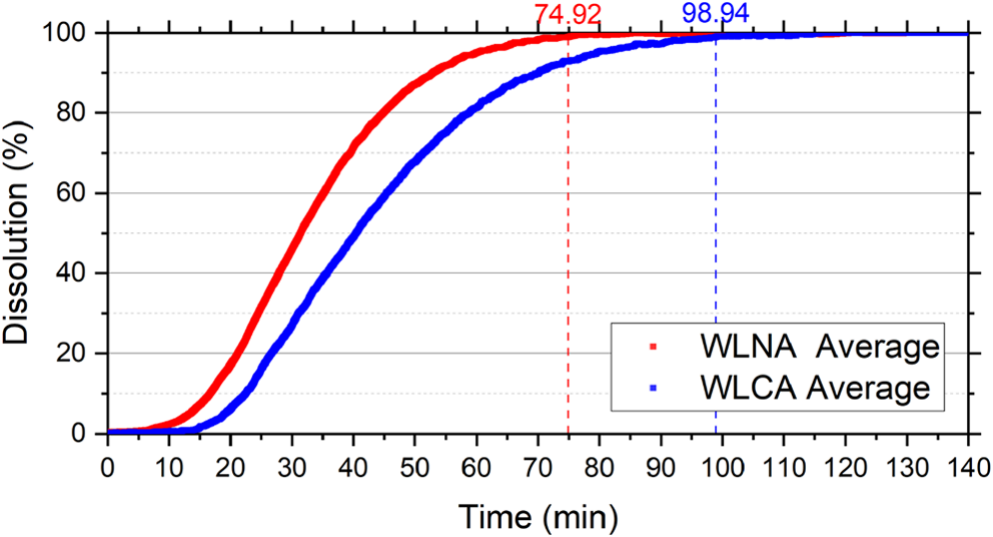
Dissolution reaction curves of Wisconsin Dolomite in 15 wt % HCl with and without additives as a function of time. WLNA corresponds to Wisconsin Dolomite reacted with 15 wt % HCl without additives, and WLCA corresponds to Wisconsin Dolomite reacted with 15 wt % HCl containing corrosion inhibitor and emulsion preventer. Curves represent average values obtained from duplicate experiments.

**Figure 10:**
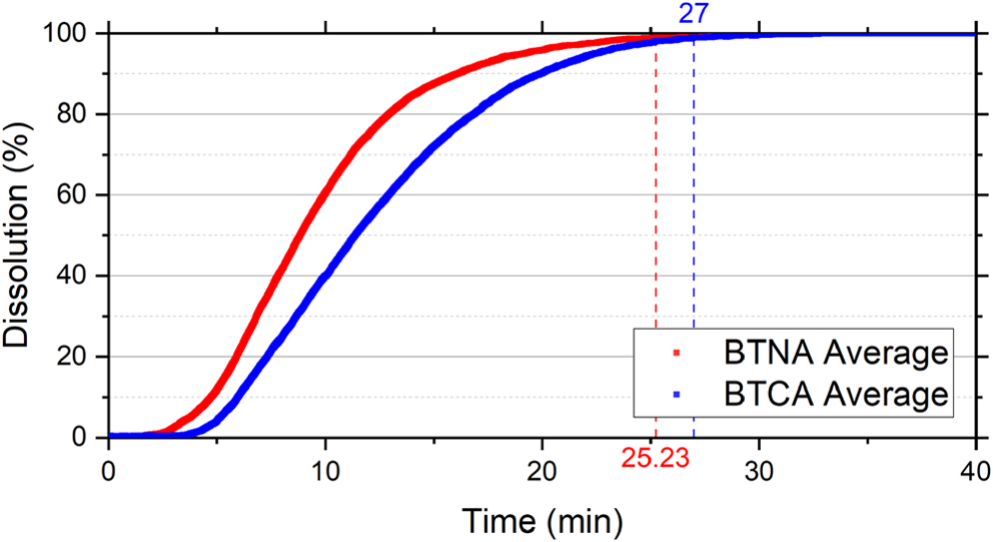
Dissolution reaction curves of Bonneterre Dolomite in 15 wt % HCl with and without additives as a function of time. BTNA corresponds to Bonneterre Dolomite reacted with 15 wt % HCl without additives, and BTCA corresponds to Bonneterre Dolomite reacted with 15 wt % HCl containing corrosion inhibitor and emulsion preventer. Curves represent average values obtained from duplicate experiments.

In addition to the graphs, the same data are detailed in Tables 7 and 8, providing more concrete numerical values to support interpretation. Table 7 highlights specific stages of the dissolution process, such as the initial reaction (1%), intermediate stages (10%, 50%, and 90%), and the final stage (99%), offering greater precision. With these data, the reaction time at each stage can be visualized more clearly, along with the corresponding standard deviations, since all tests were performed in duplicate.

**Table 7:** Average Dissolution Time of Indiana Limestone and Mount Gambier Rocks^a^

	average time (min)			
dissolution (%)	ILNA	ILCA	MGNA	MGCA
1	0.07 ± 0.02	0.57 ± 0.01	0.06 ± 0.02	0.25 ± 0.01
10	0.15 ± 0.02	1.27 ± 0.03	0.11 ± 0.01	0.32 ± 0.24
50	0.45 ± 0.07	4.93 ± 0.18	0.23 ± 0.02	4.12 ± 0.87
90	1.23 ± 0.03	13.67 ± 0.20	0.51 ± 0.01	12.52 ± 2.28
99	2.02 ± 0.01	36.28 ± 1.63	0.70 ± 0.01	20.07 ± 3.21

a IL: Indiana Limestone; MG: Mount Gambier Limestone. NA: acid without additives; CA: acid containing corrosion inhibitor and emulsion preventer.

**Table 8:** Average Dissolution Time of Wisconsin Dolomite and Bonneterre Dolomite Rocks^a^

	average time (min)			
dissolution (%)	BTNA	BTCA	WLNA	WLCA
1	2.47 ± 0.32	3.85 ± 0.13	6.67 ± 1.22	13.92 ± 0.26
10	4.72 ± 0.17	5.95 ± 0.04	16.60 ± 0.01	22.35 ± 0.76
50	8.83 ± 0.26	11.37 ± 0.02	31.32 ± 1.10	40.35 ± 1.93
90	16.05 ± 2.20	19.92 ± 0.23	52.83 ± 1.24	70.09 ± 4.67
99	25.23 ± 3.83	27.00 ± 0.06	74.92 ± 2.43	98.94 ± 3.07

a WL: Wisconsin Dolomite; BT: Bonneterre Dolomite. NA: acid without additives; CA: acid containing corrosion inhibitor and emulsion preventer.

The reactions occur significantly more slowly in the WL and BT samples compared to IL and MG, both at the beginning and at the end of the process. This behavior is consistent with the lithological nature of the rocks. According to,^48^ the dissolution of BT (CaMg­(CO_3_)_2_) might occur in two stages: first, HCl reacts with dolomite, releasing Ca^2+^ and forming solid MgCO_3_; then, the remaining MgCO_3_ reacts with H^+^, releasing Mg^2+^. Similar preferential dissolution of Ca-bearing phases and the formation of Mg-rich residual structures have been observed using X-ray CT imaging during acid–rock interactions, supporting the mechanisms discussed here.^49^ This stepwise mechanism makes the dissolution of BT slower than that of rocks composed predominantly of CaCO_3_, which react directly with HCl in a single step, thus retarding the kinetics relative to IL and MG, which are mainly composed of CaCO_3_. In addition, the WL sample presents high SiO_2_ content, a mineral with low reactivity in acidic media containing HCl.^50^ As previously discussed, in the IL and MG samples, the presence of additives led to a more pronounced reduction in dissolution kinetics compared to dolomitic rocks, confirming that mass-transfer-controlled systems are more sensitive to surfactant-induced retardation mechanisms.

These results provide valuable information for designing acid stimulation strategies in carbonate reservoirs. Understanding the role of mineralogical composition and the influence of additives enables the fine-tuning of acid formulations according to the specific conditions of each reservoir.

In formations containing significant amounts of dolomite or siliceous minerals, slower reaction rates may indicate the need for longer contact times or modified acid formulations to ensure effective matrix stimulation. Additionally, different acid pumping strategies may be required, such as adjusting the injection rate to control the acid-rock reaction front and optimize penetration into the reservoir.

In calcite-rich formations, excessively fast acid–rock reactions can lead to rapid acid consumption near the wellbore, resulting in severe face dissolution and limited penetration of the stimulating fluid.^39,40^ Several studies conducted in different regions, including the United States, China, and Saudi Arabia, have demonstrated that high calcite contents can render conventional 15 wt % HCl spearhead treatments ineffective, as the acidity is rapidly exhausted within the first few millimeters of the rock matrix, leading to poor development of effective porosity and permeability.^31,51,52^ Accordingly, the use of additives can help control the reactivity of HCl, reducing near-wellbore acid spending and favoring the formation of deeper wormholes. This effect is expected to become even more relevant under high-temperature reservoir conditions, although such conditions were not evaluated in the present study. As a next step, acid flow experiments will be conducted to determine the pore volume to breakthrough (PV_bt_) using different acid compositions, additive systems, and temperatures, to validate and quantify the observed improvement in stimulation efficiency.

## 4 Conclusions

The results demonstrated that the dissolution behavior of hydrochloric acid in carbonate rocks is strongly influenced by mineralogy, pore structure, and the presence of surfactant-based additives. Predominantly calcitic rocks, such as IL and MG, exhibited faster dissolution governed by the mass transfer of H^+^ ions, whereas dolomitic rocks, such as WL and BT, displayed a distinct behavior, characterized by slower dissolution controlled by the surface reaction.

The addition of a corrosion inhibitor and an emulsion preventer significantly affected the reaction kinetics, slowing the dissolution rate in all samples. This effect was more pronounced for the calcitic rocks, likely due to foam formation and the reduction in H^+^ availability at the rock surface, phenomena associated with the presence of surfactants in the additive formulations. The dolomitic rocks, in turn, were less affected by this change in the interfacial environment.

Furthermore, petrophysical characterization revealed relevant structural differences among the samples, with variations in porosity and pore-size distribution directly influencing the reactivity of the acid system. Rocks with higher porosity and a greater proportion of megapores, such as MG, exhibited stronger interaction with the acid, whereas formations with lower porosity and fewer megapores, such as IL, showed more limited reactivity.

For calcitic formations, the additives showed potential to retard reactivity and promote deeper acid penetration, which may represent a direct benefit for field-scale stimulation efficiency.
